# Simultaneous determination of mometasone furoate and calcipotriol in a binary mixture by validated HPLC and chemometric-assisted UV spectrophotometric methods and identification of degradation products by LC-MS

**DOI:** 10.22038/IJBMS.2022.65436.14396

**Published:** 2023-01

**Authors:** Maryam Jahani, Maryam Akaberi, Tahereh Heidari, Hossein Kamali, Mojgan Nejabat, Omid Rajabi, Farzin Hadizadeh

**Affiliations:** 1 Department of Pharmaceutical Control, School of Pharmacy, Mashhad University of Medical Sciences, Mashhad, Iran; 2 Department of Pharmacognosy, School of Pharmacy, Mashhad University of Medical Sciences, Mashhad, Iran; 3 Department of Chemistry, Faculty of Sciences, Ferdowsi University of Mashhad, Mashhad, Iran; 4 Department of Pharmaceutics, School of Pharmacy, Mashhad University of Medical Sciences, Mashhad, Iran; 5 Department of Medicinal Chemistry, School of Pharmacy, Mashhad University of Medical Sciences, Mashhad, Iran; 6 Biotechnology Research Center, Pharmaceutical Technology Institute, Mashhad University of Medical Sciences, Mashhad, Iran

**Keywords:** Calcipotriol, HPLC, Mometasone furoate, Spectrophotometric, Stress study, Validation

## Abstract

**Objective(s):**

A new binary mixture containing mometasone furoate (MF) and calcipotriol (CP) is suggested to manage psoriasis; since the combined stability profile of these drugs is poorly understood.

**Materials and Methods:**

Herein MF, CP, and their mixtures were subjected to various stress conditions. Also, stability-indicating HPLC was developed and validated according to ICH guidelines with Box-Behnken design. The degradation products (DPs) were predicted *in silico* and identified using LC-MS. The bioactivity and toxicity of DPs were studied using molecular docking and alamarBlue assay, respectively. Spectroscopic techniques of the first derivative, first-derivative ratio, and the mean-centering of ratio spectra were also used to determine MF and CP in the mixture because of spectra overlapping.

**Results:**

The major degradants for MF in alkaline conditions were DP1, DP2, and DP3, while in thermal and UV conditions, only DP1 was generated. CP gave one degradant in all conditions. No new impurity was observed in the MF and CP mixtures. The results of spectrophotometry showed good linearity in the range of 4-50 and 2-20 µg/ml, while linearity for HPLC was in the range of 4–50 and 0.5–2.5 µg/ml for MF and CP, respectively. Recovery was 99.61–100.38% for UV and 100.4% for HPLC methods of MF and 100.6–101.4% for UV and 99.5% for HPLC methods of CP.

**Conclusion:**

The developed methods can be used as simple, accurate, precise, and rapid techniques for routine quality control of MF and CP mixtures.

## Introduction

**Figure 1:**
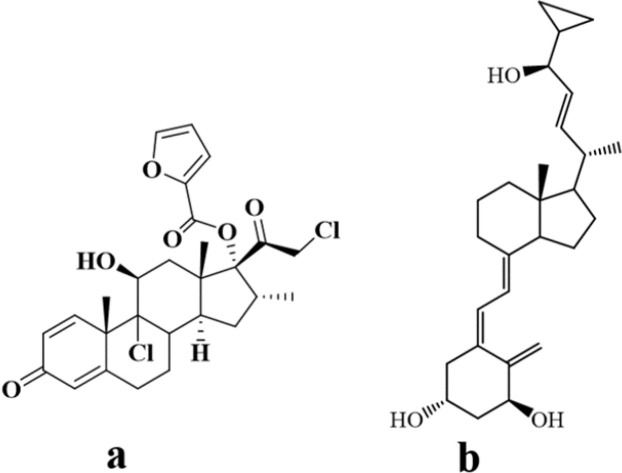
Structures of (a) mometasone furoate and (b) calcipotriol

Psoriasis, a chronic disease affecting 1–3% of the world’s population, is characterized by erythema, scaling, and inflammatory infiltration (1-3). Among topical treatments, corticosteroids such as mometasone furoate (MF) and vitamin D analogs such as calcipotriol (CP) (Figures 1a & 1b) are recommended (4-6). Combination therapy with CP and a corticosteroid result in additive clinical effects and reduced skin irritation (7-9). Several commercial products of the fixed-dose combination (FDC) of CP and betamethasone dipropionate (BD) are present in the market. Clinically, MF has some benefits compared with BD (6, 10), since it has a relatively strong anti-inflammatory potency, low systemic side effects, and rapid onset of action (11, 12), but the combination of CP and MF has not been reported yet.

According to ICH Q8R2 and FDA guidelines, the stability of FDC products needs to be evaluated (13). What generally remains to be understood about a new combination is producing new impurities (14). Forced degradation studies are used to understand the intrinsic stability of active pharmaceutical ingredients (APIs) and pharmaceutical products for degradation pathways and degradation products (DPs) information (15, 16). In addition, stress testing is suggested to demonstrate stability-indicating and specificity methods. Previously, several individual HPLC runs were performed to separate APIs and preservatives in the same finished formulation. However, developing a single run for analyzing every finished product is becoming more common. A single HPLC run can be developed by design expert software employing statistical concepts to compute the simultaneous effects of critical parameters (17, 18). Although different techniques are used for analyzing all components, such as HPTLC, HPLC, and spectrophotometric methods, the UV spectrophotometric method is preferred due to its simplicity and low cost. The main problem in the quantitative determination of APIs by the spectrophotometric method is the overlapping spectra of components in mixtures. Hence, many methods such as first-derivative spectrophotometry (^1^D) (19), first-derivative ratio (^1^DD) (20, 21), and mean-centering ratio (MCR) (22) have been applied to manipulate the interference spectra. 

Some studies have investigated the stability, degradation pathways, and DPs of MF (23-29) and CP (30-32) individually or in combination. However, no spectrophotometric method has been reported for the quantitative determination of CP, and there is little information identifying its DPs. This study aimed to evaluate the stability of a dosage form of MF and CP for future clinical studies. The capability of HPLC, ^1^D, ^1^DD, and MCR for simultaneous analysis of both drugs in combination without any preliminary separation steps was also evaluated. DPs were identified using LC-MS and predicted by the Zeneth and Fukui indices. Furthermore, the activity and toxicity of DPs were studied through molecular docking and alamarBlue assay, respectively.

## Materials and Methods

***Chemicals and Reagents***

We purchased the primary reference standard of MF, mineral oil, white beeswax, and propylene glycol from Kish Medipharm Pharmaceutical Co. (Iran), the primary reference standard of CP from Euroasia’s Company (India), penicillin-streptomycin (Pen-Strep) and fetal bovine serum (FBS) from Gibco (USA), Dulbecco’s Modified Eagle Medium (DMEM) and alamarBlue from Sigma-Aldrich (USA), and NIH/3T3 cell line from the Pasteur Institute (Iran). Ultra-pure water was obtained from a TKA-GenPure water purification unit (Germany), and HPLC analytical grade methanol, phosphate buffer salt, hydrochloric acid (37%), sodium hydroxide, and hydrogen peroxide (30%) were obtained from Merck (Germany).

***Computational studies***

The mechanism of MF and CP degradation was predicted by density functional theory (DFT) calculations performed with the Gaussian 16 W program package (www.gaussian.com). The B3LYP functional with a 6-31G (d, p) basis set was applied to optimize the geometries of MF and CP and all intermediates and products. The Fukui function predicted the electrophilic and nucleophilic sites (33). Degradation of MF and CP under oxidative, hydrolysis (acidic and alkaline), and photolysis conditions were predicted by Zeneth version 7 with Knowledge Base Z2016.1.1.mdb (Lhasa Limited, Leeds, UK). AutoZeneth mode was performed with processing constraints set for pH 1–13 at 50–80 °C, and photolytic and oxidative susceptibility with step set to 50.

To study the bioactivity of degradation products of MF, the structure of MF in complex with glucocorticoid receptors was taken from a pdb data bank with an action code of 4p6w. All structures of MF and its DPs were drawn and optimized in MOE2019 (www.chemcomp.com) with an Amber10 EHT forcefield. Docking was performed in MOE 2019, location of MF in complex with glucocorticoids receptor in x-ray structure was considered as an active site. We used default settings for MOE2019. 

***Preparation of*** ***solutions***

*Stock* *and working-standard solutions*

Standard stock solutions of MF and CP (each, 2 mg/ml) alone and in combination were prepared by dissolving the compounds in methanol and sonicating the solutions (Soniprep 150 UK) for 15 min. The standard solutions were stored at -20 °C. Fresh working-standard solutions of 100 μg/ml from MF and 40 μg/ml from CP were prepared.

*Standard solutions for UV-VIS and HPLC methods*

Fifteen solutions containing different ratios of CP (2–20 µg/ml) and MF (5–40 µg/ml) were prepared by diluting working standards (Table S1). The absorption spectra of solutions were measured by UV spectrophotometric analysis (Shimadzu, Japan, Model UV 1800) in the range of 200–400 nm.

For HPLC, solutions containing different ratios of 0.5–2.5 µg/ml of CP and 5–40 µg/ml of MF were prepared in methanol from their relative stock solutions.

*Sample preparation*

The samples for the determination of CP and MF in ointment formulation (3.2 g) were prepared according to a previously published method (22). An in-house ointment formulation (3.2 g) containing mineral oil, white beeswax, propylene glycol, MF (1 mg/g), and CP (0.05 mg/g) was weighed and dispersed in 8 ml of MeOH. The mixture was heated in a water bath at 50 °C for 5 min until melted completely. The tube was then kept in the refrigerator for 30 min, after which it was centrifuged for 10 min at 4000 rpm, and 5 ml of the MeOH layer was transferred to a 50 ml volumetric flask made up of MeOH. Then, 5 ml of the final solution was transferred to two different 10 ml volumetric flasks. One was completed to the volume with MeOH to get a solution claimed to involve 20 μg/ml of MF and 1 μg/ml of CP. This solution was used in the HPLC determination method. 1D, 1DD, and MCR used spiking as enrichment techniques for spectrophotometry. The other flask was used after being spiked with 1 ml of CP working solution (40 μg/ml), then completed to the volume with MeOH to have a solution containing 20 μg/ml of MF and 5 μg/ml of CP. The declared concentration of CP in the preparation was calculated after subtracting the added concentration (standard solution of CP 4 μg/ml analyzed by using the same procedure). A solution of blank ointment treated by the same process was used as a blank in the HPLC and spectrophotometric methods. The concentrations of the drugs were selected in their linearity ranges.

*Preparation of degraded samples of MF and CP*

MF, CP, and their binary mixture were subjected to stress in variable conditions and different times of exposure, temperature, and strength to obtain 10–20% degradation according to ICH guidelines (34). For the hydrolytic degradation test, 5 ml of the standard solutions (2 mg/ml in methanol) were diluted with 5 ml of a stressor (described in Table S2) and kept at 60 °C for 30–72 hr. For oxidative degradation, 5 ml of 30% (v/v) H_2_O_2_ was added to 5 ml of the methanol stock solutions, and the samples were kept at room temperature for 4 hr. The photolytic degradation of MF and CP were studied by exposing their solutions (100 µg/ml) to 2 × ICH recommended light illumination unit consisting of 3 fluorescent lights with cold white light (standard illuminant D65, 6.500K), and 2 UV-lamps in the spectral range of 320–400 nm (1.2 million lux hr + 200 W h m-2) for 72 hr and 30 min, respectively. A thermal degradation study was carried out by exposing the stock solution samples to different temperatures (25 and 60 °C) for 1, 4, and 24 hr.

***Chemometric-assisted UV-VIS spectrophotometric methods***

^1^ *D method*

The first-order derivative spectra were obtained in the range of 210–310 nm (Δƛ=1 nm). The wavelengths of 265 nm (zero-crossing of CP) and 247 nm (zero-crossing of MF) were used for spectrophotometric measurements of MF and CP, respectively.

^1^ *DD method*

Ratio spectra obtained by the normalized spectrum of each compound as a divisor generated a constant value for its concentration along with the whole spectra. The normalized spectrum of each compound was obtained by using the MATLAB program. Based on the first derivative of ratio spectra, this method was found using Δƛ=1 nm (35). The recorded absorbance of the laboratory-prepared mixtures (Table S1) and the extracted ointment was divided by the normalized spectrum of CP, then the first derivative spectra were found for the new spectra produced taking Δƛ1 nm, then the concentration of MF was calculated from the amplitude at 272 nm using the corresponding regression equation. The concentration of CP was calculated by the same procedure, except that the divisor was the normalized spectrum of MF and the determination was carried out at 270 nm using the corresponding regression equation.

*MCR method*

This method is based on the mean-centering of ratio spectra (22). The scanned spectra were exported to MATLAB for calculation, then the spectra of the prepared mixture (210–300 nm) were divided by the normalized spectrum of CP, and the ratio spectra of MF were obtained and mean-centered. Also, the spectra of the prepared mixture and the extracted ointment (220–270 nm) were divided by the normalized spectrum of MF, and the ratio spectra of CP were obtained and mean-centered. The calibration curves for CP and MF were constructed by plotting the mean-centered values at 239 nm and 270 nm, respectively, versus their corresponding concentrations, and the regression equations were computed.

***Optimization of chromatographic conditions using chemometric design***

For optimization of the HPLC (LC-20AD system, with the SPD-M20A photodiode array detector (DAD), Shimadzu, Japan), the Box-Behnken design model (BBD) was performed by Design-Expert software (v12.0) (36). Preliminary trials were carried out using various values for chromatographic conditions such as ƛ, flow rate, and organic modifier concentration (methanol). These critical parameters at two levels were investigated for the desirability of optimized conditions: flow rate in the range of 0.7–1.3 ml/min, ƛ of 247-264 nm, and methanol content in the range of 80–90% v/v. Responses such as resolutions (R), retention times of MF (t_R_ MF) and CP (t_R _CP), and area under the curves (AUC) of MF and CP were investigated. A total of 12 runs were performed as given in the BBD (Table S3). A solution containing 20 µg/ml of MF and 1 µg/ml of CP was used for all the experimental runs performed per the selected experimental design.

***Method validation***

Analytical methods were developed and validated based on the ICH Q2(R1) guideline (37) to demonstrate that the proposed methods were suitable for their intended purpose. The validation characteristics of the proposed processes, including specificity, linearity, limit of quantification (LOQ), limit of detection (LOD), precision, and accuracy, were determined. The RP-HPLC method was also validated for system suitability and robustness.

***System suitability***

According to USP, the system suitability for HPLC was carried out using six replicate injections of 20 µg/ml and 1 µg/ml standard solutions of MF and CP, respectively. The chromatographic parameters (capacity factor, theoretical plates, resolution, and tailing factors) were determined and compared with reference values.

***Specificity***

Specificity is the method’s ability to accurately measure the analyte response in the presence of all potential sample components. Complete separation of MF from CP and DPs with acceptable peak shapes and without any apparent shoulders can confirm the specificity of this method.

***Linearity***

For the spectrophotometric method, a line was plotted between suitable concentrations and peak amplitudes. All solutions were prepared in the range of 2–20 µg/ml of CP and 5–40 µg/ml of MF. For the HPLC method, a line was plotted between the peak area of MF and CP, as calculated from the chromatogram, and suitable concentrations prepared in the range of 0.5–2.5 µg/ml of CP and 5–40 µg/ml of MF solutions containing different ratios.

***LOD and LOQ***

LOD and LOQ were calculated based on the standard deviation (SD) of the response and the slope, using 3.3 σ/s and 10 σ/s equations, respectively 

LOD$=\frac{\mathrm{SD y}-\mathrm{intercept}}{\mathrm{slope of the calibration curve}}\times 3.3$ (1)

LOQ=$\frac{\mathrm{SD y}-\mathrm{intercept}}{\mathrm{slope of the calibration curve}}\times 10$ (2)

***Precision, accuracy, and assay***

Accuracy was reported as the percentage of recovery by determining concentration levels of 80, 100, and 120% of MF and CP in samples. The concentrations were obtained from the corresponding regression equations. The precision was reported by determining the relative standard deviation (RSD) of inter-day and intra-day precisions of four different concentrations of MF and CP each performed in triplicate.

The proposed methods were applied to assay the amount of MF and CP in the extracted ointment samples. The assay results were calculated based on mean recovery. The UV-Vis spectrophotometry method for the determination of CP was calculated after subtracting the added standard concentrations. The standard solution of CP (4 µg/ml) was analyzed using the same procedure.

***Robustness***

The robustness was determined by deliberately changing experimental conditions to study their effect on responses. The three parameters, including the percentage of methanol (85% ± 2) of the mobile phase, ƛ (254 nm ±2), and the flow rate (1.0 ml/min ± 0.1) were selected. Examined responses were theoretical plates, resolution, tailing factors, resolution and AUC of MF and CP (assay), and t_R_ of MF and CP.

***Identification of MF and CP DPs by LC-MS***

LC-MS/MS was carried out using the Agilent HPLC (1200 series, USA) system connected to an Agilent 6410 MS/MS triple quadrupole mass spectrometer equipped with an electrospray ion source (ESI). The samples were examined using a full-scan positive mode. Nitrogen gas at a pressure of 45 psi was used as a nebulizer. Data processing was carried out using the MassHunter software package to identify the molecular mass of the degradation products of MF and CP. The chromatographic separation was achieved using a Teknokroma tracer excel column (C8 250×4.6 mm, 5 µm), isocratic mode (85:15% v/v MeOH: H_2_O), and flow rate of 1 ml/min. 

***In vitro cytotoxicity***

The cytotoxicity of MF and CP under forced degradation conditions was studied using the alamarBlue test (38). The optimal concentrations of CP and MF for application to NIH/3T3 cells were determined. The cells were exposed to a series of methanol solutions of CP (1.25–10 µg/ml) and MF (1.25–5 µg/ml). Neither CP nor MF showed any cytotoxicity at 2.5, 5, and 1.25, 2.5 µg/ml, respectively. Therefore, these concentrations were selected for further evaluation. Briefly, 100 μl of fibroblast NIH/3T3 cell suspension was added to each well of a 96-well plate (DMEM culture medium containing 10% FBS and 0.1% Pen-Strep) with a density of 6×10^3^ cells per each well and incubated for 24 hr. After incubation, 100 μl of fresh medium containing samples were added to achieve final concentrations of 2.5 and 1.25 μg/ml of MF and MF exposed to alkaline conditions, heat, and light, and 5 and 2.5 μg/ml of CP and CP exposed to heat, light, and acidic conditions. NIH/3T3 cells in contact with samples were incubated again for 24, 48, and 72 hr. To evaluate cell viability, 20 μl of alamarBlue was added to each well and incubated for 3 hr. The reduction of resazurin (blue) to fluorescent resorufin (pink) was measured using a microplate reader (Biotek, Epoch) at 600 nm. Untreated cells served as negative control and wells containing alamarBlue with the culture medium served as blank. The percentage of viable cells was calculated. These experiments were performed in triplicate.

## Results

***Spectrophotometric methods***

**Figure 2:**
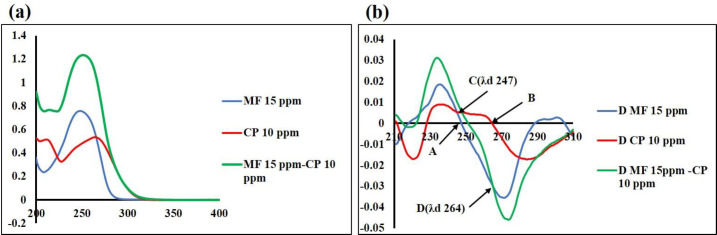
(a) The zero-order spectra of MF (blue), CP (red), and their mixture (green); (b) first-derivative spectra of MF (blue), CP (red), and their mixture (green). Points A and B are zero crossings of MF and CP, respectively. In the pure sample and mixture, points C and D show the same peak amplitude for CP and MF

MF: Mometasone furoate; CP: Calcipotriol

Chemometric-assisted UV spectrophotometer techniques developed for simultaneous determination of MF and CP specifically, ^1^D, ^1^DD, and MCR showed complete overlap (Figure 2a). 

^1^ ***D method***

The first-order deviation of CP showed zero crossing at wavelength 264 nm where MF had some absorption (Figure 2b), whereas MF showed zero crossing at wavelength 247 nm where CP had good peak amplitude (Figure 2b). In addition, Figure 2b showed the same peak amplitude for both pure standards and mixture solutions. Different concentrations of CP (2–20 µg/ml) and MF (5–40 µg/ml) were tested and the regression equations were calculated. The results showed the best recovery percentages in laboratory-prepared mixtures of the extracted ointment (Table 1). 

^1^ ***DD method***

First-derivative-ratio spectrophotometry was developed by Salinas *et al*. (39). In this study, normalized spectra of CP and MF are calculated using the MATLAB software package and used as divisor spectra thereafter (34). Δ*λ* of 1 nm was used, and the best results regarding accuracy and precision were obtained (Table 1). The derivative of the ratio spectra was used to calculate the contribution of the interfering component as equal to zero. The derivative of the ratio spectra corresponds to the amount of MF and CP (Figures S1a and b). Different concentrations of CP (2-20 µg/ml) and MF (5-40 µg/ml) in prepared mixtures and the extracted ointment were tested. ^1^DD values of CP and MF showed good linearity and accuracy at *λ* 270 and 272 nm, respectively. The concentrations were calculated using the regression equations (Table 1).

***MCR method***

MATLAB software package uses MCR methods (21), such as^1^DD, to calculate the ratio spectra and the mean-centering step. Thus, the effect of one component was eliminated in the mixture, enabling the analyst to determine the other component effects. The mean-centered spectra of different concentrations of CP (2–20 µg/ml) and MF (5–40 µg/ml) were tested (Figures S2a and b). MCR values of CP and MF showed good linearity and accuracy at 270 nm and 239 nm, respectively. The concentrations were calculated using the regression equations (Table 1).

The HPLC method also measured all samples to confirm MF and CP concentrations in the mixtures (Table S1). Results were compared using the Dunnet one-way ANOVA test. It can be concluded that the proposed spectrophotometric analytical methods are sufficiently accurate (Table S4). According to the statistical outcomes, there was no significant difference between spectrophotometric and HPLC methods (*P*-value>0.05).

***HPLC method development***

**Figure 3:**
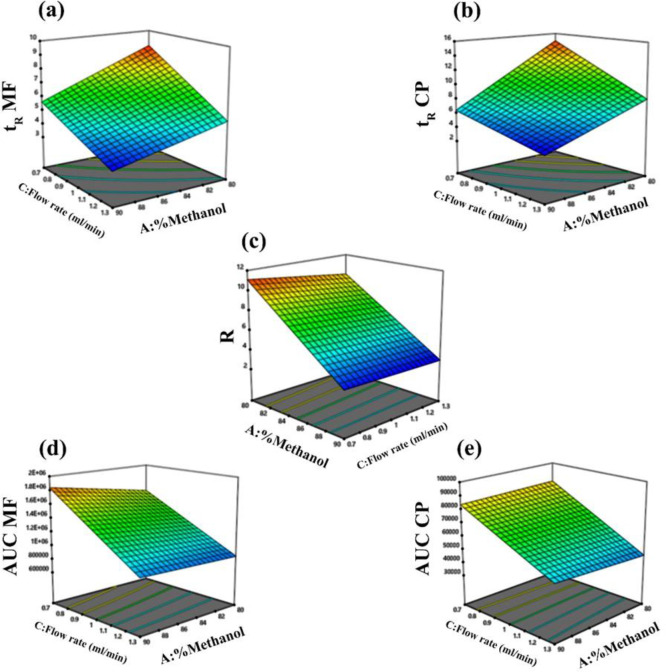
Response surface models showing the effect of methanol percentage and flow rate on the (a) retention time of MF (tR MF), (b) retention time of CP (tR CP), (c) resolution (R), (d) AUC of MF, and (e) AUC of CP

MF: Mometasone furoate; CP: Calcipotriol

HPLC method was optimized by MeOH (as the organic portion of the mobile phase) and C8 column (as the stationary phase). The chromatographic condition was developed by BBD (Design-Expert 12 software) which suggested best-fit models (*P*-values, parameter coefficients, adjusted and predicted R^2^ values, and model F values) based on the results. The percentage of MeOH, ƛ, and flow rate were investigated as factors in two levels and were studied by multivariate analysis using BBD. R1 (**t**_R_ MF), R2 (**t**_R_ CP), R3 (AUC of MF), R4 (AUC of CP), and R5** (**resolution(R)**) **were selected as five response factors described in Equations (3), (4), (5), (6), and (7) (Supplementary information). *P*-value and F-value of each factor were compared and the critical effect of this comparison on the response factors was evaluated (Table S5). In all responses, the predicted R2 value was very close to the adjusted R2 value, indicating satisfactory fitting of the model (as the differences were less than 0.2). Further, the *P*-values were less than 0.05 for all parameters. The F-value of each factor is compared and evaluated for the critical effect on the result. Therefore, the 2FI model was exhibited to be significant for the responses of MF and CP. Response surface models (Figures 3a, b, c, d, and e) clearly showed the effect of the percentage of MeOH and flow rate on responses. All results for the F-value of each intercept are shown in Table S4. F-value indicated that the percentage of organic solution in column (A) can affect resolution. The optimized method condition was methanol and water (85:15, v/v) for the mobile phase, a flow rate of 0.96 ml/min, and a detection wavelength of 254 nm. The observed data are presented in Table S6.

***Stress studies for MF and CP and identification of the DPs***

**Figure 4:**
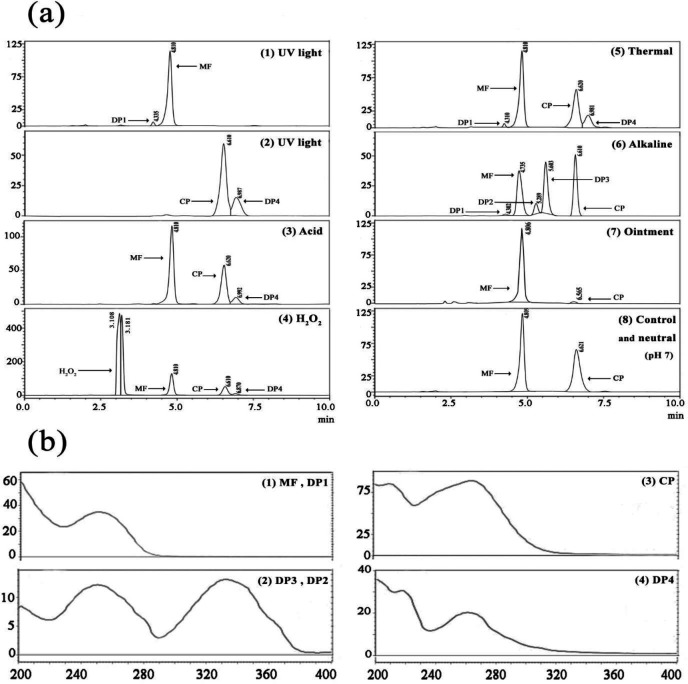
(a) Chromatographic separation of (1) MF and its DPs in a UV light chamber, (2) CP and DP in a UV light chamber, (3) separation of a binary mixture of MF, CP, and their DPs in acidic (0.001 M HCl), (4) H_2_O_2_ (15%), (5) thermal (60 °C), (6) alkaline (0.0001 M NaOH), (7) ointment (MF20 ppm/CPM1 ppm), and (8) control, neutral (water and pH 7), using the proposed HPLC method; (b) UV spectra of MF, CP, and their DPs

MF: Mometasone furoate; CP: Calcipotriol

**Figure 5:**
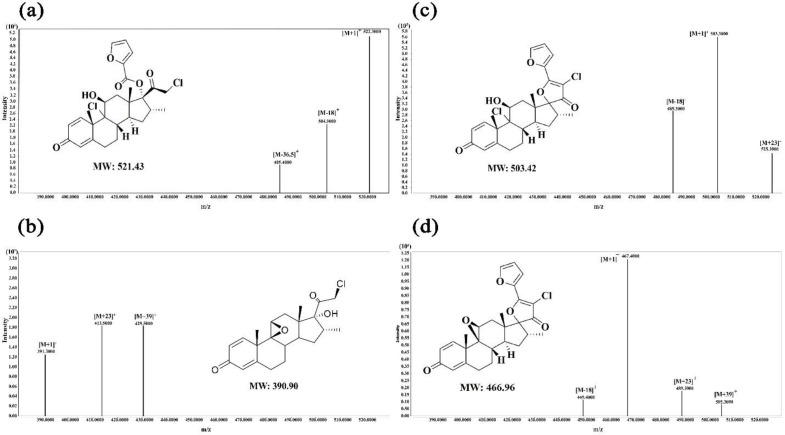
LC-MS spectra and structure of (a) standard MF, (b) DP1, (c) DP2, and (d) DP3 in ESI positive mode

A comprehensive stability study was carried out for MF, CP and their mixtures were assessed under different stress conditions (Figure 4a). The optimized HPLC method was used to detect and quantify MF and CP. The DPs were identified by LC-MS/MS and HPLC-DAD and predicted by the Zeneth and Fukui indices. Three DPs (DP1, DP2, and DP3) were observed in the current study for MF in alkaline conditions. In thermal and UV conditions, minor degradation of MF occurred, and only DP1 was observed. The retention times of DP1, DP2, and DP3 were 4.30, 5.28, and 5.60 min, respectively, and they were well separated from MF with a retention time of 4.8 min (Figure 4a6). Based on the ESI^+ ^mass spectra (Figure 5), the molecular weights of MF, DP1, DP2, and DP3 were 521.43, 390.453, 503.42, and 466.92, respectively. The molecular formulas of DP2 and DP3 agreed with the general structures C_27_H_28_Cl_2_O_5_ and C_27_H_27_ClO_5_, respectively. Similar to previous studies, the presence of DP2 and DP3 was confirmed by LC-MS analysis (23-25). An epoxide formation and HCl loss were found in alkaline conditions followed by hydrolysis of furoate moiety leading to the formation of DP1 (C_22_H_27_ClO_4_). Interestingly, the formation of the hydrolysis product was predicted by the Zeneth software (Table S7) and observed in the thermal and UV conditions for the first time in our study. The Fukui indices represented the most electrophilic (f_k_^−^), nucleophilic (f_k_^+^), and radical (f_0_) attack sites of the atoms on MF molecules. C-64 (in Fukui, all atoms in a molecule including carbons, hydrogens, oxygens, etc., are numbered altogether) was the most favorable site for nucleophilic attack by hydroxyl ions in alkaline media, leading to hydrolysis (Table S8, Figure S3a). Figure S4 shows a plausible degradation pathway for MF in alkaline conditions. In this mechanism, MF is first converted into DP2, which is subsequently converted into DP3. Dehydration on the C-17 side-chain appears. Then, a stereospecific nucleophilic attack of the 11b-hydroxyl group triggers the departure of the 9-chloro, leading to the formation of DP3 (9b, 11b-epoxide derivative). DP3 is considered the major DP in alkaline conditions (40, 41). The UV spectrum of MF shows absorption at *λ*_max_ of 247 nm due to 1,4-diene-3-keto conjugation of ring-A (Figure 4b2). The UV spectrum derived by HPLC-DAD for DP1 was similar to MF, but DP2 and DP3 had maximum wavelengths of 247 nm and 330 nm, respectively. This is consistent with the well-supported finding that other glucocorticoids have similar UV absorption at *λ*_max_ of 246 nm. This result is very interesting from a photochemical point of view because it bears two spatially separated chromophores. A cyclohexadienone moiety in ring-A and a carbonyl group at C-20 (42). It might be due to a ring produced in the side chain, resulting in a new chromophore between the carbonyl at C-20 and the neighboring double bond. Thus, the UV spectra of DP2 and DP3 exhibited two peaks at 247 nm and 330 nm (Figure 4b2).

In contrast to MF, few studies have reported fragmentation patterns and degradation pathways of CP. Only one DP (DP4) was generated. The HPLC-DAD chromatograms of solutions of CP under various temperature and stress situations are shown in Figure 4a. While CP eluted at a retention time of 6.621 min, the t_R_ of DP4 was 6.77 min. The production of DP4 was dependent on temperature (Figure 4a5). The UV spectrum of CP showed maximum absorption at 264 nm, while the UV spectra derived by HPLC-DAD of DP4 exhibited a shift to 260 nm (Figure 4b3, b4). According to UV and LC-MS fragmentation patterns, DP4 was identified as pre-Calcipotriene (pre-calci) and observed as a DP at various stress conditions. Table S8 and Figure S3b show the condensed Fukui functions for calcipotriol. As the Fukui data show, C-27 had a large f_k_^+^ and hence acted as a nucleophilic site center. According to the proposed mechanism, the first rotation around C-19-C-20 occurs, then the hydride from C-13 attacks the C-27 nucleophilic site, and pre-calci is formed (Figure S5). LC-MS/MS analyses were also performed for CP. The MS profile of CP showed the protonated molecular ion at m/z of 413 (M+H)^+^, the loss of one water molecule from the protonated molecular ion at m/z of 395 (M+H-H_2_O)^+^, and the sodium and potassium adduct ions at m/z of 435 (M+Na)^+^ and 451 (M+K)^+^, respectively (43, 44). The mass spectrum of DP4 showed similar ions to those of CP (Figure S6a). The MS/MS fragmentation pattern of the molecular ion of CP (Figure S6b) showed fragmented ions at m/z of 328, 372, and 88, similar to the fragmentation pathway of vitamin D (45). 

The LC-MS chromatograms did not show any additional peaks of DPs formed by the interaction of MF and CP (Figure S7).

***Method validation***

According to the ICH Q2 (R1), HPLC and UV methods are suitable for their intended purposes. The validation characteristics of the proposed processes, including specificity, linearity, LOQ, LOD, precision, and accuracy were determined (Table 1). The system suitability parameters were calculated through six replicate injections of 20 and 1 µg/ml of MF and CP solutions, respectively (Table S9). Robustness was evaluated by making deliberate but small changes in UV wavelength, flow rate, and methanol percentage (Table S10). The estimated responses were not significantly affected by the variation of the specified factors, indicating that this method was robust. This study showed high resolution and no chromatographic interference of the DPs with standard solutions (Figure 4a). 

***Cell viability and docking studies***

**Figure 6:**
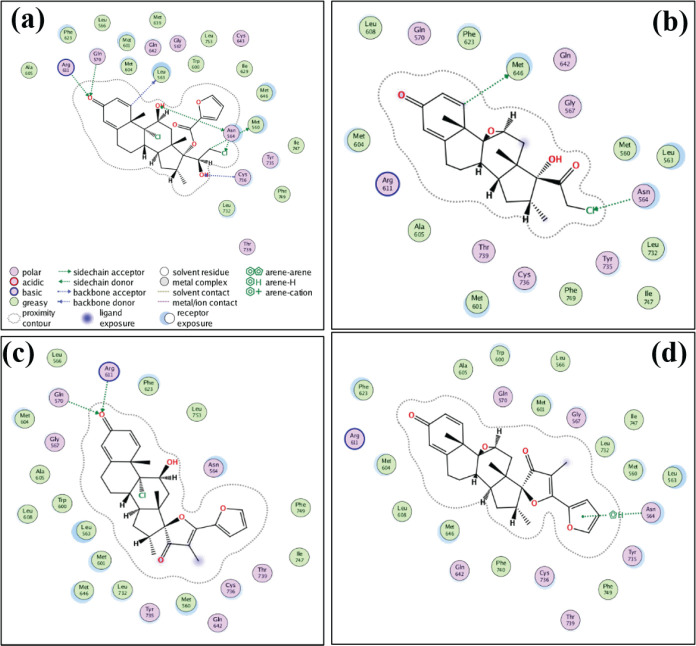
Docking of (a) MF, (b) DP1, (c) DP2, and (d) DP3 at the active site of the glucocorticoid receptor (pdb id 4p6w)

The cell viability of fibroblast NIH/3T3 treated with MF, CP, and their DPs were examined to evaluate toxicity and biocompatibility. The results were expressed as the percentage of cell-growth inhibition. The toxicity of MF, CP, and their DPs were investigated on normal fibroblast cells as skin models. According to the cell-viability value, MF and its UV and thermal DPs showed no toxicity. Alkaline degradations of MF showed significant toxicity by inhibiting 30% of cell growth, whereas MF showed 3% of growth inhibition. Thus, DP2 and DP3 only showed toxicity in alkaline conditions (Figure S8). Similar behavior was seen in another study on the A549 (human lung cancer) cell line (41). DP1, 2, and 3 showed less interaction based on docking studies. However, DP2, which had interaction with Arg670 and Gln511, showed non-hydrogen bond interactions with the receptor, and it was predicted that these products would be less effective than MF, which had interaction with Leu563, Asn 564, Met560, Gln570, Arg611, Asn564, and Cys763 (Figure 6). CP DP4 (pre-calci) under forced degradation also showed less cytotoxicity or none. Chemicals that have cell viability above 80% are considered safe and biocompatible. Figure 6. Docking of (a) MF, (b) DP1, (c) DP2, and (d) DP3 at the active site of the glucocorticoid receptor (pdb id 4p6w).

**Table 1:** Regression parameters and results of MF and CP determination by the proposed methods

Parameter	MF				CP			
	D	DD	MCR	HPLC	D	DD	MCR	HPLC
Wavelength (nm)	264	272	239	254	247	270	270	254
Linearity range (µg/ml)	5-40	5-40	5-40	5-40	2-20	2-20	2-20	0.5-2.5
Slope	0.001782	0.0147	0.189	63217	0.0005	0.0394	0.245	45192
Intercept	0.00212	0.0267	0.1369	49826	0.0001	0.0035	-0.00059	1817.4
Regression coefficient (R^2^)	0.998	0.998	0.998	0.999	0.999	0.999	0.999	0.999
^a^Accuracy	99.31±1.88	100.38±2.62	99±1.52	100.4±1.36	101±2.27	100.6±1.266	101.4±2.35	99.5±1.77
^b^Assay	100.62±2.01	98.77±1.62	102.29±2.30	101±2.54	98.01±1.8	97.77±2.22	103.5±2.3	100±1.69
^a^Intra-day RSD	1.48	1.49	0.265	0.816	2.2	1.259	2.0	1.52
^a^Inter-day RSD	1.89	1.46	1.2	1.3	2.194	1.8	2.4	1.1
^c^LOD (µg/ml)	0.856	0.343	0.325	0.3	0.110	0.07	0.106	0.0116
^d^LOQ (µg/ml)	2.569	1.03	0.976	0.704	0.33	0.21	0.320	0.0348

aAccuracy and intra-day and inter-day precision were carried out by mean recovery of concentrations: 16, 20, and 24 µg/ml and 4, 5, and 6 µg/ml of MF and CP, respectively, using the spectrophotometric method, and 16, 20, and 24 µg/ml and 0.8, 1, and 1.2 µg/ml of MF and CP, respectively, using the HPLC method (n = 3). bAssay ointment samples were prepared in triplicate (mean±SD) cLOD: limit of detection; dLOQ: limit of quantification

MF: Mometasone furoate; CP: Calcipotriol

## Discussion

Although the spectrophotometry method was economic and eco-friendly, it had the disadvantage of overlapping peaks in the UV spectra of CP and MF. Herein, ^1^DD, ^1^D, and MCR techniques were used to eliminate the absorption effect of another analyte. This work aimed to develop simple and novel spectrophotometric techniques to determine both CP and MF simultaneously in their binary mixture and ointment without preliminary separation. To the best of our knowledge, no previous data existed on the simultaneous determination of CP in the presence of other drugs using a spectrophotometric method. There is a large difference in the ratios of other drugs and CP in combination formulations leading to a deviation from Beer’s law due to the electrostatic attraction between ions (21). This problem is usually raised upon analysis of CP in the presence of MF in a solution mixture or ointment (1CP/20MF). This interference from CP could be eliminated after applying the sample enrichment techniques, such as standard addition in which a fixed amount of CP standard is added to each sample and its concentration is subtracted before calculating the claimed concentration of the drug thereafter. This technique has been used to solve the same problem in the analyses of other drug mixtures as well (22, 46). Thus, the sample enrichment technique is suitable and would resolve this problem by eliminating all types of error, that can result in a high degree of accuracy of analytical signals. As mentioned earlier, the overlapping UV spectra problem for the combination of CP and MF was solved using ^1^DD, ^1^D, and MCR techniques that eliminated the absorption effect of co-analytes and excipients. The main disadvantage of ^1^DD and MCR spectrophotometry is their multiple manipulating steps such as finding a suitable concentration for the divisor, devising, and finally calculating the derivative and mean center. In this study, normalized UV spectra of CP and MF were calculated using the MATLAB software package and used as divisor spectra thereafter (35). All the techniques used were found to be simple, accurate, precise, and rapid. In addition, the excipient compounds of the ointment did not show any interference with the absorption of the drugs, so the accuracy of the methods was higher than 99%. 

To optimize HPLC conditions, different mobile phases such as acetonitrile and MeOH were tested. MeOH was chosen as the organic portion of the mobile phase because acetonitrile did not provide adequate separation due to significant tailing. Most articles have used MeOH as the organic mobile phase for the determination of CP (30, 32). As an example, the separation of vitamin D_3_ from its isomerization impurities was good when the gradient mobile phase reached to 90% MeOH ratio, whereas the use of acetonitrile resulted in poor separation (47). In this study, the C8 column was used for the separations since it is less hydrophobic than the C18 column leading to weaker bonding reactions with the ointment matrix (long-chain alkane) and shorter t_R_s for CP and MF. The HPLC method condition was optimized by BBD such that the resolution and AUC were selected as maximum values and retention time as minimum values. The results showed that the retention time can be influenced by MeOH and flow rate; however, the flow rate has affected the t_R_ of MF more in comparison with MeOH. The interaction between the organic percentage ratio and the flow rate has influenced the retention time more than the resolution. AUC was directly related to the flow rate, decreasing from 1.3 to 0.7 ml/min, but ƛ showed a very slight effect on it.

Although the accuracy and precision of HPLC and spectrophotometric methods showed no significant difference (*P*>0.05), the HPLC method had more sensitivity and lower LOQ. So, both UV and HPLC methods can also be used for the routine determination of MF and CP in release testing.

A comprehensive stability study was carried out according to ICH Q1A guidelines for both MF and CP under different stress conditions. This helped us to develop a stability-indicating HPLC method for the simultaneous determination of MF and CP mixture. All the key DPs were well identified using the newly developed HPLC-DAD and MS. To calculate Fukui indices and predict DPs, Zeneth and DFT software were utilized. The results showed that MF was more labile in alkaline conditions than in neutral or acidic conditions. Three DPs (DP1, DP2, and DP3) were observed for MF in alkaline conditions, whereas minor degradation of MF occurred in thermal and UV conditions, and only DP1 was observed, which was also confirmed by Zeneth and DFT. The structures of DP2 and DP3, identified by LC-MS and HPLC-DAD, were different from the structures proposed by Teng *et al*. (23) who hypothesized a furoate-ester migration between C-17 and C-21 positions. While ester migration is not likely to happen in MF as a steroid compound with a halogen atom at C-21, this phenomenon has been reported for other steroids such as beclomethasone monopropionate, betamethasone-17-valerate, and hydrocortisone butyrate (see Figure S4) (48-51). CP could not tolerate the acidic condition, and it was unstable at temperatures higher than 50 °C, and very sensitive to light. According to HPLC-DAD data, LC-MS fragmentation patterns, and Zeneth and DFT, CP had one significant DP that was pre-calci (DP4) in all conditions. Previous studies have reported a similar phenomenon for vitamin D2 and ecalcidene (52, 53). There was no new degradation when MF and CP were used simultaneously under stress conditions and were stable at neutral pH. According to the cytotoxicity study, MF and its UV and thermal degradants showed no toxicity. However, toxicity was observed in alkaline degradation, and docking studies confirmed the ineffectiveness of degradation products of MF. Degradation products of CP under forced degradation did not show cytotoxicity.

## Conclusion

HPLC and chemometrics spectrophotometric methods were developed and validated for the simultaneous determination of MF and CP mixtures and ointment. The methods were found to be simple, accurate, precise, and rapid. The accuracy and precision of HPLC and spectrophotometric methods showed no significant difference (*P*>0.05), but the HPLC method had more sensitivity and lower LOQ. In conclusion, the proposed spectrophotometric method can be used as an alternative method to determine both MF and CP in commercial samples and release tests. Moreover, this combination product was compatible and can be preformulated in a topical dosage form at pH 7.

## Authors’ Contributions

MJ Helped with project administration and funding acquisition; MA Provided investigation, validation, and visualization; T H contributed by investigation and visualization; HK and MN Provided resources, writing & editing; FH Helped with conceptualization, methodology, resources, investigation, validation, and writing the original draft & editing; OR Helped with conceptualization, methodology, investigation, resources, data curation, software, validation, visualization, formal analysis, writing the original draft & editing.

## Funding

This work was supported by Mashhad University of Medical Sciences, Mashhad, Iran (grant no. 961598). 

## Conflicts of Interest

None.

## References

[1] Semenov YR, Herbosa CM, Rogers AT, Huang A, Kwatra SG, Cohen B (2021). Psoriasis and mortality in the US: Data from the national health and nutrition examination survey. J Am Acad Dermatol.

[2] An L, Li J, Liu B, Hui J, Zhang Q, Zhang X (2022). Knockdown of TRPM7 attenuates apoptosis and inflammation in neonatal necrotizing enterocolitis model cell IEC-6 via modulating TLR4/NF-κB and MEK/ERK pathways. Iranian IJBMS.

[3] Aslankoc R, Savran M, Doğuç DK, Sevimli M, Tekin H, Kaynak M (2022). Ameliorating effects of ramelteon on oxidative stress, inflammation, apoptosis, and autophagy markers in methotrexate-induced cerebral toxicity. IJBMS.

[4] Lawton S (2018). Psoriasis presentations and potential treatment pathways. Practice Nursing.

[5] Gual A, Pau-Charles I, Molin S (2016). Topical treatment for scalp psoriasis: Comparison of patient preference, quality of life and efficacy for non-alcoholic mometasone emulsion versus calcipotriol/betamethasone gel in daily clinical practice. J Dermatol Treat.

[6] Elmets CA, Korman NJ, Prater EF, Wong EB, Rupani RN, Kivelevitch D (2021). Joint AAD–NPF Guidelines of care for the management and treatment of psoriasis with topical therapy and alternative medicine modalities for psoriasis severity measures. J Am Acad Dermatol.

[7] Kleyn EC, Morsman E, Griffin L, Wu JJ, Cm van de Kerkhof P, Gulliver W (2019). Review of international psoriasis guidelines for the treatment of psoriasis: Recommendations for topical corticosteroid treatments. J Dermatol Treat.

[8] Ma L, Yang Q, Yang H, Wang G, Zheng M, Hao F (2016). Calcipotriol plus betamethasone dipropionate gel compared with calcipotriol scalp solution in the treatment of scalp psoriasis: A randomized, controlled trial investigating efficacy and safety in a Chinese population. Int J Dermatol.

[9] McCormack PL (2011). Calcipotriol/betamethasone dipropionate. Drugs.

[10] Svensson Å, Reidhav I, Gisslén H, Nordin P, Giös I (1992). A comparative study of mometasone furoate ointment and betamethasone valerate ointment in patients with psoriasis vulgaris. Curr Ther Res.

[11] Corazza M, Virgili A, Toni G, Borghi A (2018). Mometasone furoate in the treatment of vulvar lichen sclerosus: Could its formulation influence efficacy, tolerability and adherence to treatment?. J Dermatol Treat.

[12] Shinde G, Desai P, Shelke S, Patel R, Bangale G, Kulkarni D (2020). Mometasone furoate-loaded aspasomal gel for topical treatment of psoriasis: formulation, optimization, in vitro and in vivo performance. J Dermatol Treat.

[13] FDA/ICH (2006). Guidance for industry. Q8 Pharmaceutical development.

[14] Xu Q (2019). Advancing USP compendial methods for fixed dose combinations: A case study of metoprolol tartrate and hydrochlorothiazide tablets. J Pharm Anal.

[15] Davoodi J, Majidi S, Jahani M, Tayarani-najaran Z, Golmohammadzadeh S, Kamali H (2021). Implementation of design of experiments for optimization of forced degradation conditions and development of a stability-indicating HPLC method for sepiwhite. J Sep Sci.

[16] Jahani M, Fazly Bazzaz BS, Akaberi M, Rajabi O, Hadizadeh F (2021). Recent Progresses in analytical perspectives of degradation studies and impurity profiling in pharmaceutical developments: An updated review. Crit Rev Anal Chem.

[17] Tome T, Žigart N, Časar Z, Obreza A (2019). Development and optimization of liquid chromatography analytical methods by using AQbD principles: Overview and recent advances. Org Process Res Dev.

[18] El-Sayed HM, Hashem H (2020). Quality by design strategy for simultaneous HPLC determination of bromhexine HCl and its metabolite ambroxol HCl in dosage forms and plasma. Chromatographia.

[19] Panchale WA, Bakal RL (2021). First-order derivative spectrophotometric estimation of gemifloxacin mesylate and ambroxol HCl in tablet dosage form. GSC Biol Pharm Sci.

[20] Victor N, Elfiky H, Badawey A, Abdelghany M (2021). Simultaneous determination of xipamide and triamterene by first derivative, ratio difference, and derivative ratio spectrophotometric methods. Arch Pharm Sci Ain Shams univ.

[21] Essam HM, Saad MN, Elzanfaly ES, Amer SM (2021). Optimization and validation of eco-friendly RP-HPLC and univariate spectrophotometric methods for the simultaneous determination of fluorometholone and tetrahydrozoline hydrochloride. Acta Chromatogr.

[22] Lotfy HM, Salem H, Abdelkawy M, Samir A (2015). Spectrophotometric methods for simultaneous determination of betamethasone valerate and fusidic acid in their binary mixture. Spectrochim Acta A Mol Biomol Spectrosc.

[23] Teng XW, Cutler DC, Davies NM (2003). Degradation kinetics of mometasone furoate in aqueous systems. Int J Pharm.

[24] Sahasranaman S, Tang Y, Biniasz D, Hochhaus G (2005). A sensitive liquid chromatography–tandem mass spectrometry method for the quantification of mometasone furoate in human plasma. J Chromatogr B.

[25] Teng XW, Foe K, Brown KF, Cutler DJ, Davies NM (2001). High-performance liquid chromatographic analysis of mometasone furoate and its degradation products: Application to in vitro degradation studies. J Pharm Biomed Anal.

[26] Ramzia I, Marwa A, Manal A, Enas H (2013). Derivative, derivative of the ratio spectrophotometric and stability-indicating RP–HPLC methods for the determination of mometasone furoate and miconazole nitrate in cream. J Chem Pharm Res.

[27] Shaikh KA, Patil AT (2013). Stability-indicating HPLC method for the determination of mometazone furoate, oxymetazoline, phenyl ethanol and benzalkonium chloride in nasal spray solution. J Trace Anal Food & Drugs.

[28] Shaikh S, Muneera M, Thusleem O, Tahir M, Kondaguli AV (2009). A simple RP-HPLC method for the simultaneous quantitation of chlorocresol, mometasone furoate, and fusidic acid in creams. J Chromatogr Sci.

[29] Roy C, Chakrabarty J (2013). Stability-indicating validated novel RP-HPLC method for simultaneous estimation of methylparaben, ketoconazole, and mometasone furoate in topical pharmaceutical dosage formulation. Int Sch Res Notices.

[30] Singh M, Charde R, Charde MM (2015). Determination of Calcipotriene, its forced degradation and impurity analysis by HPLC. Int J Pharm Life Sci.

[31] Roy C, Chakrabarty J, Ratti RR (2013). Development and validation of a stability-indicating NP-HPLC method for simultaneous determination of betamethasone dipropionate and calcipotriene in topical dosage form. Appl Sci Res.

[32] Badilli U, Amasya G, Özkan S, Tarimci N (2013). Simultaneous determination of clobetasol propionate and calcipotriol in a novel fixed dose emulgel formulation by LC-UV. Chromatographia.

[33] Ayers PW, Parr RG (2000). Variational principles for describing chemical reactions: the Fukui function and chemical hardness revisited. J Am Chem Soc.

[34] Rignall A (2017). ICHQ1A (R2) stability testing of new drug substance and product and ICHQ1C stability testing of new dosage forms. ICH quality guidelines: an implementation guide..

[35] Mohamed EH, Lotfy HM, Hegazy MA, Mowaka S (2017). Different applications of isosbestic points, normalized spectra and dual wavelength as powerful tools for resolution of multicomponent mixtures with severely overlapping spectra. Chem Cent J.

[36] Ganorkar A, Gupta K (2017). Analytical Quality by Design: A mini review. Biomed J Sci Tech Res.

[37] Guideline ICH (2005). Validation of analytical procedures: Text and Methodology. Q2 (R1)..

[38] Kumar P, Nagarajan A, Uchil PD (2018). Analysis of cell viability by the alamarBlue assay. Cold Spring Harbor Protocols.

[39] Salinas F, Nevado JB, Mansilla AE (1990). A new spectrophotometric method for quantitative multicomponent analysis resolution of mixtures of salicylic and salicyluric acids. Talanta.

[40] Sahasranaman S, Issar M, Toth G, Horváth G, Hochhaus G (2004). Characterization of degradation products of mometasone furoate. Die Pharmazie-An Inter J Pharm Sci.

[41] Vichare V, Choudhari VP, Reddy MV (2019). Study of intrinsic stability of mometasone furoate in presence of salicylic acid by HPTLC and characterization, cytotoxicity testing of major degradation product of mometasone furoate. Curr Pharm Anal.

[42] Iqbal J, Gupta A, Husain A (2006). Photochemistry of clobetasol propionate, a steroidal anti-inflammatory drug. Arkivoc.

[43] Simonsen L, Høy G, Didriksen E, Persson J, Melchior N, Hansen J (2004). Development of a new formulation combining calcipotriol and betamethasone dipropionate in an ointment vehicle. Drug Dev Ind Pharm.

[44] Li X, Wang J, Li G, Lin C, Zhang X, Sun Y (2013). Evaluation of calcipotriol transdermal permeation through pig, rat and mouse skin using liquid chromatography–tandem mass spectrometry. Biomed Chromatogr.

[45] Napoli JL, Koszewski NJ, Horst RL (1986). (14) Isolation and identification of vitamin D metabolites. Meth Enzymol.

[46] Harvey D ( 2000). Modern analytical chemistry.

[47] Mahmoodani F, Perera CO, Fedrizzi B, Abernethy G, Chen H (2017). Degradation studies of cholecalciferol (vitamin D3) using HPLC-DAD, UHPLC-MS/MS and chemical derivatization. Food chemistry.

[48] Bundgaard H, Hansen J (1981). Studies on the stability of corticosteroids VI Kinetics of the rearrangement of betamethasone-17-valerate to the 21-valerate ester in aqueous solution. Int. J. Pharm..

[49] Foe K, Cheung HA, Tattam BN, Brown KF, Seale JP (1998). Degradation products of beclomethasone dipropionate in human plasma. Drug metabolism and disposition.

[50] Yip Y, Po ALW, Irwin W (1983). Kinetics of decomposition and formulation of hydrocortisone butyrate in semiaqueous and gel systems. J Pharm Sci.

[51] Mori M, Pimpinelli N, Giannotti B (1994). Topical corticosteroids and unwanted local effects. Drug safety.

[52] Zhang F, Nunes M, Segmuller B, Dunphy R, Hesse RH, Setty SKS (2006). Degradation chemistry of a Vitamin D analogue (ecalcidene) investigated by HPLC–MS, HPLC–NMR and chemical derivatization. J Pharm Biomed Anal.

[53] Bhogadi R, Satyanarayana A, Rao NS, Arutla S, Reddy AM (2015). Stability indicating RP-HPLC method for estimation of impurities of vitamin D 3 analogue and corticosteroid used as antipsoriatic drugs An attempt to characterize pre-calcipotriene. Am J Analyt Chem.

